# COVID-19 meta-analyses: a scoping review and quality assessment

**DOI:** 10.31744/einstein_journal/2021AO6002

**Published:** 2021-03-09

**Authors:** Gabriel Natan Pires, Andréia Gomes Bezerra, Thainá Baenninger de Oliveira, Samuel Fen I Chen, Victor Davis Apostolakis Malfatti, Victoria Feiner Ferreira de Mello, Alyne Niyama, Vitor Luiz Selva Pinto, Monica Levy Andersen, Sergio Tufik

**Affiliations:** 1 Universidade Federal de São Paulo São PauloSP Brazil Universidade Federal de São Paulo, São Paulo, SP, Brazil.; 2 Faculdade de Ciências Médicas da Santa Casa de São Paulo São PauloSP Brazil Faculdade de Ciências Médicas da Santa Casa de São Paulo, São Paulo, SP, Brazil.

**Keywords:** Bibliometrics, Coronavirus, COVID-19, Meta-analysis, Betacoronavirus, SARS-CoV-2, Scientometrics, Systematic review

## Abstract

**Objective:**

To carry out a scoping review of the meta-analyses published regarding about coronavirus disease 2019 (COVID-19), evaluating their main characteristics, publication trends and methodological quality.

**Methods:**

A bibliometric search was performed in PubMed^®^, Scopus and Web of Science, focusing on meta-analyses about COVID-2019 disease. Bibliometric and descriptive data for the included articles were extracted and the methodological quality of the included meta-analyses was evaluated using A Measurement Tool to Assess Systematic Reviews.

**Results:**

A total of 348 meta-analyses were considered eligible. The first meta-analysis about COVID-19 disease was published on February 26, 2020, and the number of meta-analyses has grown rapidly since then. Most of them were published in infectious disease and virology journals. The greatest number come from China, followed by the United States, Italy and the United Kingdom. On average, these meta-analyses included 23 studies and 15,200 participants. Overall quality was remarkably low, and only 8.9% of them could be considered as of high confidence level.

**Conclusion:**

Although well-designed meta-analyses about COVID-19 disease have already been published, the majority are of low quality. Thus, all stakeholders playing a role in COVID-19 deseases, including policy makers, researchers, publishers and journals, should prioritize well-designed meta-analyses, performed only when the background information seem suitable, and discouraging those of low quality or that use suboptimal methods.

## INTRODUCTION

Since coronavirus disease 2019 (COVID-19) was recognized as a serious public health threat, researchers from all over the world have devoted a great deal of time and effort to characterize and understand this new disease, resulting in an unprecedented surge in the number of publications.^(^^1^^–^^3^^)^ On average, more than 200 new articles about COVID-19 have been indexed in PubMed^®^, every day.^(^^4^^)^

All stakeholders involved in academic research and scientific publishing have directed efforts to enable an efficient publication outflow: governments and supporting agencies are releasing special funding and grants for research about COVID-19; ethics committees and other regulatory agencies are prioritizing COVID-19-related projects;^(^^5^^)^ publishers are applying open access policies to COVID-19 articles; and journals are reviewing COVID-19 articles using fast-track processes.^(^^6^^,^^7^^)^

All these efforts have the ultimate goal of enhancing knowledge and generating evidence about COVID-19. From an evidence-based perspective, meta-analyses are usually regarded as the experimental approach that generates the highest level of scientific evidence. Thus, not surprisingly, meta-analyses regarding COVID-19 are already being published. Prompted by this remarkable and constant growth in publication output, a discussion in respect of the quality and ethical standards of these articles has already begun.^(^^6^^,^^8^^–^^10^^)^

Both the need to release and publish data rapidly, and the shortened peer-review times, can result in a reduction in the quality of the published reports. Since meta-analyses are also subjected to same publication environment, this reduction in quality might also be true for them. In order to quantify the problem and foresee potential drawbacks in the evidence synthesis of COVID-19 research, it is important to quantify the amount of meta-analyses being published, their characteristics, their methods and the average quality of these reports.

## OBJECTIVE

To carry out a scoping review of the meta-analyses published regarding coronavirus disease 2019, evaluating their main characteristics, publication trends and methodological quality.

## METHODS

A bibliometric search was performed in PubMed^®^, Scopus and Web of Science, to retrieve all meta-analyses published relating to COVID-19. The search strategy, which was initially designed for PubMed^®^ and adapted to other databases, is described below. It was performed on August 18, 2020 and no publication filters were used. The protocol used was registered in the Open Science Framework (https://osf.io/tnps2/).

Search was performed using the following terms: (meta-analysis [publication type] OR meta-analysis as topic [mesh] OR meta-analysis [tiab] OR meta-analyses [tiab] OR meta-analysis [tiab] OR metaanalyses [tiab] OR meta-analysis [tiab] OR metanalyses [tiab]) AND (covid-19 [supplementary concept] OR covid-19 OR covid19 OR “novel coronavirus” OR “sars-cov-2” OR “2019-ncov”).

The resulting articles were screened and evaluated in a two-step process. In the first, titles and abstracts were reviewed by two independent authors. Discrepancies were resolved by consensus. Next, the full texts of the articles selected in the first step were evaluated by a single author per article and double-checked by a second reviewer. Only meta-analyses dealing with COVID-19-related issues were considered eligible. Regarding the PI(E)CO strategy, COVID-19 could be addressed as an intervention (I), exposure (E) or outcome (O), while there was no restriction regarding the population (P) or comparators (C). Six main exclusion criteria were applied: articles published before 2019; protocols of systematic reviews and meta-analyses; research designs other than meta-analyses (systematic reviews with no meta-analyses were excluded); meta-analyses not related to COVID-19; meta-analyses for which data came from sources other than previously published articles (*e.g*., meta-analyses of geographical or meteorological data), and articles for which the full text could not be found. Meta-analyses in which COVID-19 data were considered concurrently with other diseases, such as the severe acute respiratory syndrome (SARS) or the Middle East respiratory syndrome (MERS), were also considered eligible.

Data extraction was performed by a single author per article. The following data was extracted: bibliometric information (author, publication year, publication date, journal and country – based on the first affiliation of the first author), number of articles and individuals included in the meta-analyses, pre-publication registration (*e.g*., International Prospective Register of Systematic Reviews – PROSPERO), databases screened and research designs considered eligible (only randomized controlled trials (RCT), only non-randomized designs or both). The articles were classified into the same 13 categories used by PROSPERO to categorize COVID-19-related protocols of systematic reviews. Finally, the methodological quality of the included meta-analyses was evaluated using A Measurement Tool to Assess Systematic Reviews (AMSTAR 2.0).^(^^11^^)^All authors applying tool had undergone training with a senior reviewer, and the evaluation criteria of each item were discussed and standardized in a meeting. To assure consistency and accuracy in data abstraction, two rounds of calibration were performed: in the first, all the authors involved in data extraction analyzed a set of ten articles from the sample. In the second round, a sample of 130 articles were evaluated by pairs of reviewers. Reviewers could start data extraction and quality assessment independently, only when consistency was assured.

All data were analyzed qualitatively, presented in a descriptive fashion when needed (percentages and absolute frequency for categorical data, and mean and standard deviation for numeric variables). This scoping review was performed in accordance with the Preferred Reporting Items for Systematic Reviews andMeta-Analyses Extension for Scoping Reviews (PRISMA-ScR) guidelines.^(^^12^^)^

The assignment of authors to each step in this systematic review is disclosed in the registered protocol (https://osf.io/tnps2/).

## RESULTS

**Figure 1:**
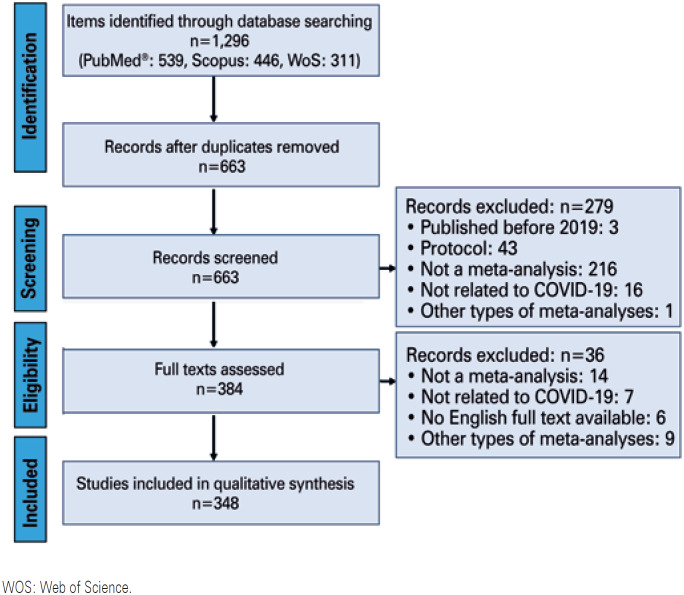
Flowchart of article identification and inclusion

**Figure 2:**
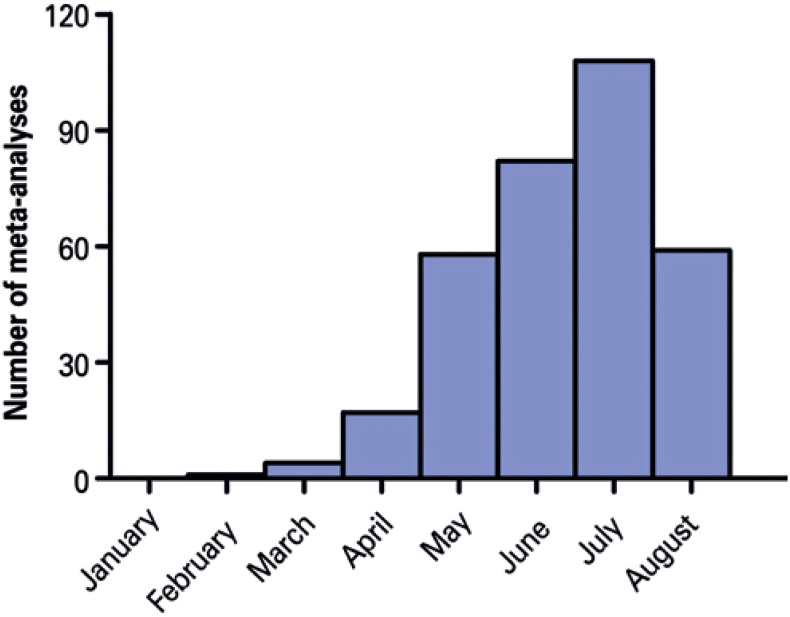
Temporal analysis of publication output. Number of meta-analyses published per month is shown since the publication of the first meta-analysis (February 26). Articles whose publication date could not be determined, or that are scheduled for September 2020 onwards are not counted (n=329). Data from August should not be interpreted as a reduction in the number of meta-analyses published, as the search strategy was made on August 18, 2020

Out of 1,296 meta-analyses, 348 were considered eligible (Figure 1). The first article in this sample was indexed on February 26, 2020.^(^^13^^)^ In this short period since the publication of this article (176 days until August 18), an average of 1.95 meta-analyses were published per day. This number has been increasing, as shown in figure 2. Among these articles, 336 (96.5%) focused only on COVID-19, while 12 (3.4%) merged COVID-19 data with data for other diseases (SARS: 12; MERS: 11; Ebola and influenza: 1). The meta-analyses included an average of 22.94±21.49 studies (median: 16) and 15,250.57±46,876.16 participants (median: 4,121).

**Figure 3:**
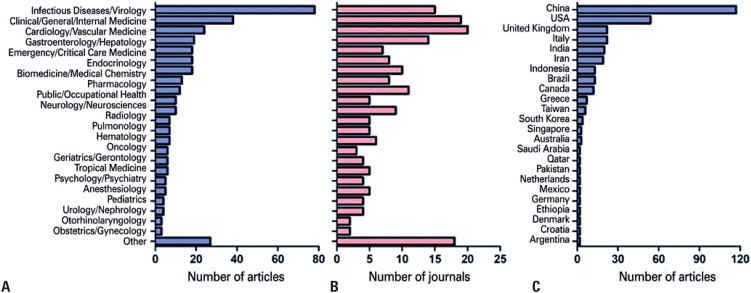
Publication output per subject area and country. (A) Number of articles on each subject area; (B) Number of journals in each subject area; (C) Number of articles per country. The time since the first local case of COVID-19, the ability to include studies published in local languages, and the recent surge in the publication of meta-analyses in general may have contributed to China being ranked as producer of the largest number of analyses, with an output double that of the United States

The studies were published in 193 different journals. The Journal of Medical Virology contributed the most, publishing 35 meta-analyses on COVID-19 (9.1%), followed by the Journal of Infection, with 19 articles (4.9%). Eight journals published between six and nine meta-analyses each, 39 published between two and four meta-analyses each, and the remaining 144 journals published a single meta-analysis each. Most meta-analyses were published in infectious disease or virology journals (78 articles in 15 journals), followed by clinical/internal/general medicine (38 articles in 19 journals), cardiology and vascular medicine (24 articles in 20 journals) and gastroenterology (19 articles in 14 journals) (Figures 3A and 3B).

A total of 37 countries were listed in the publication output (Figure 3C). China was the most productive country, with 117 articles (33.6%), followed by the United States with 54 articles (15.1%), and Italy and the United Kingdom, with 22 articles each (12.6%).

Only 58 meta-analyses (16.7%) registered a protocol, being PROSPERO the most commonly used protocol registry. Six meta-analyses (1.6%) included only RCT, while the remaining were based on observational studies, or on a combination of multiple research designs.

Regarding the search strategies of the meta-analyses, 107 of them (30.7%) screened five or more databases; while 23 (6.6%) screened a single database, a non-recommended practice due to the increased risk of selection bias. PubMed^®^ and MEDLINE^®^ were the most frequently employed database (342 articles; 98.3%), followed by Embase (204 articles; 58.6%), Web of Science (130 articles; 37.4%), Cochrane Central (129 articles; 37.1%), and Scopus (94 articles; 27.0%). Pre-print sources, such as medRxiv and bioRxiv, were used in 74 articles (21.3%). Regional databases from China were used in 74 articles (21.3%).

The most common focuses of the included meta-analyses were disease prognosis (200 articles; 57.5%), epidemiology (130 articles; 37.4%), diagnosis (48 articles; 13.8%), health impacts (43 articles; 12.4%) and treatments (42 articles; 12.1%).

**Figure 4:**
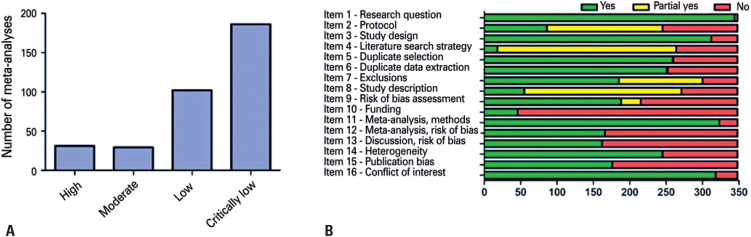
Quality assessment of meta-analyses about COVID-19 using A Measurement Tool to Assess Systematic Reviews. (A) Number of meta-analyses according to different levels of confidence; (B) Evaluation of each criterion included in A Measurement Tool to Assess Systematic Reviews. Green means that an item was adequately addressed; yellow means it was partially addressed, and red means it was not addressed

Quality assessment using AMSTAR 2.0 revealed that only 31 of the meta-analyses (8.9%) were of a quality that allowed a high overall confidence in the results of the review, while 186 (53.4%) were of a critically low level. Quality analyses according to each item in AMSTAR 2.0 are shown in figure 4.

## DISCUSSION

Meta-analyses are regarded as the highest level of evidence. Therefore, healthcare decision makers frequently rely on them for evidence-based guidance.^(^^14^^)^ In the current pandemic situation, healthcare practices and policies need to be developed in a rapid fashion, making meta-analyses an important source of evidence. However, it must be kept in mind that the quality of the meta-analyses does not rely solely on the methodology used, but also on the number and quality of the articles included. Thus, the main question to answer is: do we have enough high-quality studies to perform meta-analyses about COVID-19?.

The building blocks of meta-analyses are the studies they include. The reasonability about performing a meta-analyses depends on the number of articles being analyzed, and the robustness of its results depends on the quality of the articles included (according to the concept of “garbage in, garbage out”). Appropriate statistical methods need to be employed in meta-analyses. Indeed, increasing the amount of low-bias meta-analyses (*i.e.* those with reliable methods and robust methodology) might increase the proportion of reliable scientific findings overall.^(^^15^^)^ Although the total number of articles about COVID-19 is considerable, questions have been raised about the overall quality of the studies,^(^^6^^,^^8^^–^^10^^)^ and in respect of meta-analyses, some cases of methodologically flawed studies have already been reported.^(^^16^^,^^17^^)^

The meta-analyses included in this study comprise a fair amount of data, with an average of 23 studies and 15,000 participants per article. This is a surprising finding, since we would expect the meta-analyses published to be much smaller, given that they were published a short time after the start of the pandemic. It probably reflects the fact that epidemiological studies on COVID-19, with very large cohorts, were published relatively soon after the initial outbreak. However, although there does not seem to be lack of data, the quality of these meta-analyses is remarkably low, with only 31 (8.9%) considered to be of high quality.

It should also be noted that no meta-analysis based on RCT only has yet been published. This is explained by several meta-analyses performed on topics that are only solvable by observational studies (such as prevalence of risk factors). However, meta-analyses about themes that depend on properly performed RCT (such as pharmacological interventions and vaccines) do not seem feasible in the short term.

The sources and topics of the meta-analyses also provided interesting results. More than 50% of studies come from only four countries: China, United States, Italy and United Kingdom. Although these countries are among those most affected by COVID-19, it would be misleading to make this correlation, since they were already among the top six meta-analyses producers, even before the pandemic.^(^^18^^)^ It is interesting to note that China's production is almost twice that of the United States, probably reflecting the geometric increase in their meta-analyses publication output, as from 2009.^(^^19^^)^

Most of the meta-analyses were published in virology and infectious disease journals, addressing prognostic factors and epidemiology. This is to be expected, given that these were the first topics for which a reasonable amount of data became available. Although PROSPERO has already been registering systematic reviews about treatments and vaccines, meta-analyses on these topics seem rather unfeasible in the short term (and will be less informative if based on the limited number of completed the RCT available).

One positive aspect of the included meta-analyses is the inclusion of pre-print sources (18.0%) and national databases (mostly Chinese; 22.5%). It is estimated that about a quarter of all relevant evidence about COVID-19 is not in PubMed^®^, but might be available in pre-print sources^(^^20^^)^ and not including them represents an obvious publication bias. The same is true in respect of the inclusion of national databases, since a fair amount of data about epidemiology and clinical description are available only in Chinese.

The overall number of meta-analyses being published has increased considerably in recent years,^(^^21^^)^ and this mass publication of meta-analyses has been heavily criticized.^(^^22^^,^^23^^)^ Among the main problems relating to the increased number of meta-analyses being published are duplicated efforts, conflicting results, low quality and limited practical value.^(^^22^^)^ While our study focused on an analysis of the quality of the studies, one might expect these same problems are also present in COVID-19-related meta-analyses. One example of conflicting results in meta-analyses about COVID-19 was found in respect of smoking. A meta-analysis published in May 2020 concluded that smoking does not represent a risk factor for severe cases of COVID-19.^(^^24^^)^However, two subsequent meta-analyses highlighted methodological errors in the original analysis, and came to an opposite conclusion.^(^^16^^,^^25^^)^ Since then, several other systematic reviews have been performed in relation to smoking, including a recent meta-analysis performed using robust methods (a living review with Bayesian meta-analysis, currently in its seventh edition).^(^^26^^)^ Its conclusions reinforced the complexity of this association, since they found current smokers are at reduced risk of severe acute respiratory syndrome coronavirus 2 (SARS-CoV-2) infection, but former smokers are at increased risk of severe cases and death from COVID-19. Thus, despite the growing number of meta-analyses about COVID-19 inconsistencies, low quality and methodological weaknesses among them might reduce their potential to generate applicable findings. This is particularly important for policy-makers and health care managers, who might wish to use meta-analyses to drive their practices and policies.

The idea that meta-analyses represent the unequivocal highest level of medical evidence is outdated,^(^^27^^)^ and this affirmation can certainly not be applied to all individual meta-analyses. Weak or imprecise meta-analyses actually produce less evidence than, for instance, a well conducted large observational study or a proper multicenter randomized controlled trial. Based on these considerations and the results of our review, we make the following recommendations to different stakeholders about how to deal with meta-analyses regarding COVID-19.

To policy makers: meta-analyses are undeniably important for policy makers, so that they can design and implement policies employing on reliable evidence-based information. However, current meta-analyses about COVID-19 are of limited quality and, in some cases, have discrepant results. Thus, in these times of uncertain evidence, decision-making should not blindly rely on meta-analyses, but rather on a deep analysis of literature and the evidence it contains.

To researchers and authors of meta-analyses: new meta-analyses about COVID-19 are welcome, as long as they provide new high-quality evidence. Low-quality meta-analyses (premature, redundant, dubious, inconclusive, or performed with suboptimal practices) are counterproductive, for impairing proper evidence synthesis. Thus, we suggest that prospective authors of meta-analyses perform a serious critical appraisal about the need for their meta-analyses. The following factors should be evaluated: whether there is enough data to perform a meta-analysis; if the available data seems reliable, comparable and free from heterogeneity; whether other meta-analyses in the same field have already been published or protocols have been registered. Researchers should rely on a search of protocol registrations for systematic reviews and meta-analyses to avoid duplicated and redundant efforts. Quality and methodological robustness should not be overlooked in favor of rapid publication, or it is likely that the quality of the review will be poor (as seen in our results).

To journals and publishers: meta-analyses usually lead to a fair amount of citations and good visibility to journals publishing them.^(^^28^^)^ Thus, in the current situation, journals might feel tempted to publish meta-analyses, while ignoring faulty methods and low-quality procedures. Interestingly, as the number of articles about COVID-19 has increased, so has the number of articles being withdrawn and retracted.^(^^29^^)^Journals should comply with their social role of being a media for evidence-based science. Recent discussions have highlighted the role of journals in promoting the publication of high-quality meta-analyses, while discouraging those that do not follow optimal methodological standards.^(^^30^^)^ This is especially relevant in the current situation. Peer review can be made faster, as long as its quality and fairness are not compromised. We would suggest that a researcher with experience in meta-analyses is always included as a reviewer, or to have a statistician on the editorial board, to ensure that faulty or low-quality meta-analyses are not published.

A few points need to be considered for a proper interpretation of our study: this is a scoping review, rather than a systematic review. Thus, rather than aiming to perform a meta-analysis ourselves, our review was intended to describe a panorama about the current meta-analyses about COVID-19, focusing on the general framework of the field, instead of on the individual aspects of each study; - we decided to exclude systematic reviews with no meta-analyses. This is because our focus was on evidence synthesis, which is much reduced in reviews not including meta-analyses, and also, in many cases, the term “systematic review” is misused.^(^^31^^)^ However, an enormous amount of systematic reviews has been published, whose quality has already been appraised in a previous article.^(^^32^^)^ This is a time-restrained review. In the near future, meta-analyses on most aspects of COVID-19 will probably become viable. However, a study focusing attention on the quality of meta-analyses published so far in respect of COVID-19 is timely; and the call to prioritize attention on high-quality meta-analyses is important.

## CONCLUSION

Meta-analyses are an important tool in evidence-based medicine. The number of meta-analyses in respect of COVID-19 is increasing and will continue to rise. We believe that meta-analyses will play an important role in COVID-19, defining its epidemiological characteristics, the most suitable treatments, and supporting policy-making. However, while we do support the need for meta-analyses about COVID-19, it should not be taken as a license to produce them indiscriminately.

Although some very well-designed meta-analyses about COVID-19 have already been published, the majority published so far are of poor quality, and are likely to causes more harm than good. Few meta-analysis about COVID-19 in our review could be considered as of high quality, while more than a half were considered of critically low quality. Thus, all stakeholders playing a role in COVID-19 research and publication (including policy makers, researchers, publishers and journals) should focus on proper evidence-based research, supporting well designed meta-analyses, performed only when the background information seems suitable, and discourage those of low quality or that use suboptimal methods.
