# Supplementary material for: COVID-19 meta-analyses: a scoping review and quality assessment
Source: Einstein (Sao Paulo). 2021 Mar 9;19:eAO6002. doi: 10.31744/einstein_journal/2021AO6002 (PMC7942841; doi:10.31744/einstein_journal/2021AO6002)
Supplement: Translation

# Translation: Metanálises sobre COVID-19: revisão de escopo e análise de qualidade

**Authors:** Gabriel Natan Pires, Andréia Gomes Bezerra, Thainá Baenninger de Oliveira, Samuel Fen I Chen, Victor Davis Apostolakis Malfatti, Victoria Feiner Ferreira de Mello, Alyne Niyama, Vitor Luiz Selva Pinto, Monica Levy Andersen, Sergio Tufik

**Affiliations:** 1 São PauloSP Brasil Universidade Federal de São Paulo, São Paulo, SP, Brasil.; 2 São PauloSP Brasil Faculdade de Ciências Médicas da Santa Casa de São Paulo, São Paulo, SP, Brasil.

**Keywords:** Bibliometria, Coronavírus, COVID-19, Metanálise, Betacoronavírus, SARS-CoV-2, Cientometria, Revisão sistemática

## Abstract

**Objetivo:**

Realizar uma revisão de escopo das metanálises publicadas sobre a doença pelo coronavírus 2019 (COVID-19), avaliando suas principais características, tendências de publicação e qualidade metodológica.

**Métodos:**

Uma busca bibliométrica foi realizada em PubMed®, Scopus e *Web of Science*, com foco em metanálises sobre a doença pelo COVID-19. Foram extraídos dados bibliométricos e descritivos dos artigos incluídos, e a qualidade metodológica foi avaliada usando a ferramenta *A Measurement Tool to Assess Systematic Reviews*.

**Resultados:**

Um total de 348 metanálises foram consideradas elegíveis. A primeira delas foi publicada em 26 de fevereiro de 2020, e, desde então, o número dessas publicações cresceu rapidamente. A maioria foi publicada em periódicos de infectologia e virologia. Grande parte é proveniente da China, seguida dos Estados Unidos, da Itália e do Reino Unido. Em média, as metanálises incluíram 23 estudos e 15.200 participantes. Em geral, a qualidade metodológica foi baixa, e apenas 8,9% delas podem ser consideradas de algum grau de confiabilidade

**Conclusão:**

Embora algumas metanálises bem conduzidas sobre a doença pelo COVID-19 tenham sido publicadas, a maioria apresenta baixa qualidade. Todos os envolvidos na abordagem da doença pelo COVID-19, incluindo formuladores de políticas, pesquisadores, editoras e periódicos, devem dar prioridade a metanálises de alta qualidade, realizadas apenas quando os dados são viáveis, e desencorajar as de baixa qualidade ou conduzidas com métodos subótimos.

## INTRODUÇÃO

Desde que a doença pelo coronavírus 2019 (COVID-19) foi reconhecida como uma séria ameaça à saúde pública, pesquisadores de todo o mundo têm dedicado tempo e esforço para caracterizar e entender essa nova doença, o que acabou levando ao aumento sem precedentes no número de publicações.(1-3) Em média, mais de 200 novos artigos sobre a COVID-19 estão sendo indexados diariamente no PubMed®.(4)

Todas as partes envolvidas em pesquisa acadêmica e publicação científica trabalham para garantir a eficiên­cia do fluxo de publicação. Governos e agências de apoio oferecem financiamento e bolsas especiais para pes­quisas sobre COVID-19; comitês de ética e outros órgãos reguladores priorizam projetos relacionados à doença;(5) editoras oferecem políticas de acesso livre aos artigos sobre COVID-19, e periódicos usam processos abreviados para revisão de artigos sobre COVID-19.(6,7)

Todos esses esforços visam aumentar o conhecimento e gerar evidências sobre essa doença. De uma perspectiva baseada em evidências, as metanálises são geralmente consideradas a abordagem experimental que gera o mais alto nível de evidência científica. Assim, não é de surpreender que metanálises sobre a COVID-19 já estejam sendo publicadas. Em decorrência desse cresci­mento evidente e constante nas publicações sobre o tema, iniciou-se uma discussão sobre a qualidade e os padrões éticos desses artigos.(6,8-10)

A necessidade de divulgar e publicar rapidamente os dados, somada ao prazo mais curto da revisão por pares, pode resultar em diminuição na qualidade dos re­latórios publicados. Como as metanálises também estão sujeitas ao mesmo ambiente de publicação, essa diminuição da qualidade pode afetá-las também. Para quantificar o problema e prever possíveis desvantagens na síntese de evidências das pesquisas sobre COVID-19, é importante quantificar o número de metanálises que estão sendo publicadas, suas características, seus métodos e a qualidade média dessas publicações.

## OBJETIVO

Realizar uma revisão de escopo das metanálises publicadas sobre a doença pelo coronavírus 2019, avaliando suas principais características, tendências de publicação e qualidade metodológica.

## MÉTODOS

Foi realizada uma busca bibliométrica nas bases PubMed®, Scopus e *Web of Science*, para levantamento de todas as metanálises publicadas sobre COVID-19. A estratégia de busca, inicialmente concebida para o PubMed® e adaptada às demais bases de dados, está descrita a seguir. A busca foi realizada em 18 de agosto de 2020 e não foram utilizados filtros de publicação. O protocolo usado foi registrado no *Open Science Framework* (https://osf.io/tnps2/).

A busca utilizou os seguintes termos: *(meta-analysis [publication type] OR meta-analysis as topic [mesh] OR meta-analysis [tiab] OR meta-analyses [tiab] OR meta-analysis [tiab] OR metaanalyses [tiab] OR meta-analysis [tiab] OR metanalyses [tiab]) AND (covid-19 [supplementaryconcept] OR covid-19 OR covid19 OR “novel coronavirus” OR “sars-cov-2” OR “2019-ncov”).*

Os artigos resultantes foram triados e avaliados em um processo de duas etapas. Na primeira, os títulos e resumos foram revisados por dois autores independentes. As divergências foram resolvidas por consenso. Em seguida, o texto completo dos artigos selecionados na primeira etapa foi avaliado por um único autor por artigo e verificado por um segundo revisor. Foram consideradas elegíveis somente as metanálises que tratassem de questões relacionadas à COVID-19. Em relação à estratégia PI(E)CO, a COVID-19 poderia ser abordada como intervenção (I), exposição (E) ou desfecho (O), e não houve restrição quanto à população (P) e nem aos comparadores (C). Foram aplicados seis critérios principais de exclusão: artigos publicados antes de 2019; protocolos de revisões sistemáticas e metanálises; desenhos de pesquisa que não fossem metanálises (revisões sistemáticas sem metanálises foram excluídas); metanálises não relacionadas à COVID-19; metanálises com dados provindos de fontes que não fossem artigos publicados anteriormente (por exemplo, metanálises de dados geográficos ou meteorológicos) e artigos cujo texto completo não foi encontrado. Foram consideradas elegíveis também as metanálises em que dados da COVID-19 foram analisados junto dos de outras doenças, como síndrome respiratória aguda grave (SARS) ou síndrome respiratória do Oriente Médio (MERS).

A extração dos dados foi realizada por um único autor, por artigo. Foram extraídos os seguintes dados: informações bibliométricas (autor, ano de publicação, data de publicação, periódico e país – com base na primeira afiliação do primeiro autor), número de artigos e indivíduos incluídos nas metanálises, registro pré-publicação (por exemplo, *International Prospective Register of Systematic Reviews* – PROSPERO), bancos de dados pesquisados e desenhos de pesquisa considerados elegíveis (somente ensaios controlados randomizados – ECR –,somente desenhos não randomizados, ou ambos). Os artigos foram classificados nas mesmas 13 categorias utilizadas no PROSPERO para categorização de protocolos de revisões sistemáticas relacionados à COVID-19. Finalmente, a qualidade metodológica das metanálises incluídas foi avaliada na ferramenta *A Measurement Tool to Assess Systematic Reviews* (AMSTAR) 2.0.(11) Todos os autores que usaram essa ferramenta foram treinados por um revisor sênior, e os critérios de avaliação de cada item foram discutidos e padronizados em uma reunião. Para garantir consistência e acurácia na abstração dos dados, foram realizadas duas rodadas de calibração: na primeira, todos os autores envolvidos na extração dos dados analisaram um conjunto de dez artigos da amostra. Na segunda rodada, uma amostra de 130 artigos foi avaliada por pares de revisores. Os revisores puderam iniciar a extração dos dados e a avaliação da qualidade de forma independente apenas depois de garantir a consistência.

Todos os dados foram analisados qualitativamente e apresentados de forma descritiva, quando necessário (percentagens e frequência absoluta para variáveis categóricas, e média e desvio-padrão para variáveis numéricas). Esta revisão de escopo foi realizada de acordo com as diretrizes *Preferred Reporting Items for SystematicReviews and Meta-Analyses Extension for Scoping Reviews* (PRISMA-ScR).(12)

A atribuição de cada autor em cada etapa desta revisão sistemática está especificada no protocolo registrado (https://osf.io/tnps2/).

## RESULTADOS

Das 1.296 metanálises, 348 foram consideradas elegíveis (Figura 1). O primeiro artigo da amostra foi indexado em 26 de fevereiro de 2020.(13) No curto período desde a publicação do artigo (176 dias até 18 de agosto), em média, foi publicada 1,95 metanálise por dia. Esse número aumentou, como mostrado na figura 2. Entre esses artigos, em 336 (96,5%) se abordou apenas a COVID-19, enquanto 12 (3,4%) mesclaram dados sobre COVID-19 e outras doenças (12 sobre SARS, 11 sobre MERS, 1 sobre Ebola e 1 sobre influenza). As metanálises incluíram média de 22,94±21,49 estudos (mediana: 16) e 15.250,57±46.876,16 participantes (mediana: 4.121).

**Figura 1:** Fluxograma de identificação e inclusão de artigos

**Figura 2:** Análise temporal da produção de publicações. O número de metanálises publicadas por mês é mostrado desde a publicação da primeira delas (26 de fevereiro). Os artigos cuja data de publicação não pôde ser determinada ou previstos para publicação a partir de setembro de 2020 não foram contados (n=329). Os dados de agosto não devem ser interpretados como uma redução no número de metanálises publicadas, já que a estratégia de busca foi colocada em prática em 18 de agosto de 2020

Os estudos foram publicados em 193 periódicos diferentes. O *Journal of Medical Virology* ficou em primeiro lugar, com 35 metanálises publicadas sobre COVID-19 (9,1%), seguido pelo *Journal of Infection* com 19 artigos (4,9%). Oito periódicos publicaram entre seis e nove metanálises cada, 39 publicaram entre duas e quatro metanálises cada, e os 144 periódicos restantes publicaram uma única metanálise cada. A maioria das metanálises foi publicada em revistas de infectologia ou virologia (78 artigos em 15 revistas), seguida de clínica médica/medicina interna/geral (38 artigos em 19 revistas), cardiologia e medicina vascular (24 artigos em 20 revistas) e gastrenterologia (19 artigos em 14 revistas) (Figuras 3A e 3B).

**Figura 3:** Publicações por área temática e país. (A) Número de artigos em cada área temática; (B) Número de periódicos em cada área temática; (C) Número de artigos por país. O tempo decorrido desde o primeiro caso local de COVID-19, a capacidade de incluir estudos publicados em idiomas locais e o recente aumento geral na publicação de metanálises podem ter contribuído para a classificação da China como a maior produtora de análises, com produção duas vezes maior que os Estados Unidos

No total, 37 países figuraram na lista de publicações (Figura 3C). China foi o país com a maior produção, com 117 artigos (33,6%), seguido pelos Estados Unidos, com 54 artigos (15,1%), e Itália e Reino Unido, com 22 artigos cada (12,6%).

Apenas 58 metanálises (16,7%) registaram um protocolo, sendo o PROSPERO o registro de protocolos mais utilizado. Seis metanálises (1,6%) incluíram apenas ECR, enquanto as restantes basearam-se em es­tudos observacionais ou em uma combinação de diferentes desenhos.

Com relação às estratégias de busca das metanálises, 107 delas (30,7%) pesquisaram cinco ou mais bases de dados, enquanto 23 (6,6%) pesquisaram uma única base de dados, prática não recomendada devido ao maior risco de viés de seleção. PubMed® e MEDLINE® foram as bases de dados mais usadas (342 artigos; 98,3%), seguidas por Embase (204 artigos; 58,6%), *Web of Science* (130 artigos; 37,4%), CochraneCentral (129 artigos; 37,1%) e Scopus (94 artigos; 27,0%). Fontes pré-impressão, como medRxiv e bioRxiv, foram usadas em 74 artigos (21,3%). Bancos de dados regionais da China foram usados em 74 artigos (21,3%).

Os focos mais comuns nas metanálises incluídas foram o prognóstico da doença (200 artigos; 57,5%), epidemiologia (130 artigos; 37,4%), diagnóstico (48 artigos; 13,8%), impactos na saúde (43 artigos; 12,4%) e tratamentos (42 artigos; 12,1%).

A avaliação da qualidade utilizando o AMSTAR 2.0 revelou que apenas 31 das metanálises (8,9%) tiveram qualidade suficiente para uma alta confiança nos resultados da revisão, enquanto 186 (53,4%) tiveram nível de qualidade criticamente baixo. As análises de qualidade, de acordo com cada item do AMSTAR 2.0, são mostradas na figura 4.

**Figura 4:** Avaliação de qualidade das metanálises sobre COVID-19 usando a ferramenta *A Measurement Tool to Assess Systematic Reviews*. (A) Número de metanálises conforme os diferentes níveis de confiança; (B) Avaliação de cada critério incluído na ferramenta *A Measurement Tool to Assess Systematic Reviews.* A cor verde significa que um item foi abordado de forma adequada; amarelo significa que foi parcialmente abordado de forma adequada, e vermelho significa que não foi abordado

## DISCUSSÃO

As metanálises são consideradas como o mais alto nível de evidência. Portanto, os tomadores de decisões em saúde confiam frequentemente nelas para obter orientações baseadas em evidências.(14) Na atual pandemia, práticas e políticas de saúde precisam ser desenvolvidas rapidamente, o que torna as metanálises importante fonte de evidências. No entanto, deve-se ter em mente que a qualidade das metanálises não depende apenas da metodologia utilizada, mas também do número e qualidade dos artigos incluídos. Dessa forma, a pergunta principal é: temos estudos de qualidade suficiente para produzir metanálises sobre a COVID-19?.

As principais bases das metanálises são os estudos nelas incluídos. A razoabilidade da realização de metanálises depende do número de artigos analisados, e a robustez dos resultados depende da qualidade dos artigos incluídos (de acordo com o conceito de “*garbage in, garbage out*”). As metanálises devem empregar métodos científicos apropriados. Na verdade, o aumento do número de metanálises de baixo viés (ou seja, com métodos confiáveis e metodologia robusta) pode melhorar a proporção geral de resultados científicos confiáveis.(15) Embora o número total de artigos sobre a COVID-19 seja considerável, foram levantadas dúvidas sobre a qualidade geral dos estudos(6,8-10) e, em relação às metanálises, já foram relatados alguns casos de estudos com falhas metodológicas.(16,17)

As metanálises incluídas neste estudo apresentam quantidade razoável de dados, com média de 23 estudos e 15 mil participantes por artigo. Trata-se de uma constatação surpreendente, pois o esperado seria que as metanálises publicadas fossem muito menores, já que foram produzidas pouco tempo após o início da pandemia. Isso é explicado pelo fato de que foram publicados estudos epidemiológicos sobre COVID-19, com coortes muito grandes, relativamente pouco tempo após o surto inicial. No entanto, embora não pareça haver falta de dados, a qualidade dessas metanálises é notavelmente baixa, e apenas 31 delas (8,9%) são consideradas de alta qualidade.

Vale também ressaltar que não foi publicada nenhuma metanálise baseada somente em ECR. Isso porque várias metanálises publicadas abordaram ques­tões que só poderiam ser solucionadas com estudos observacionais (como a prevalência de fatores de risco). Porém, a produção de metanálises sobre tópicos que dependam de ECR devidamente realizados (como intervenções farmacológicas e vacinas) parece não ser viável no curto prazo.

As fontes e tópicos das metanálises também mostraram resultados interessantes. Mais de 50% dos estudos provêm apenas de quatro países: China, Estados Unidos, Itália e Reino Unido. Embora esses países estejam entre os mais afetados pela COVID-19, essa correlação pode induzir ao erro, já que figuravam entre os seis principais produtores de metanálises, mesmo antes da pandemia.(18) É interessante notar que a produção da China é quase o dobro da dos Estados Unidos, o que provavelmente reflete o aumento geométrico na produção de metanálises pela China, desde 2009.(19)

A maioria das metanálises foi publicada em perió­dicos de virologia e infectologia, abordando fatores prognósticos e epidemiologia. Isso era esperado, já que esses foram os primeiros tópicos para os quais uma grande quantidade de dados foi disponibilizada. Embora o PROSPERO já esteja registrando revisões siste­máticas sobre tratamentos e vacinas, metanálises sobre esses assuntos, no curto prazo, parecem bastante inviáveis (e serão menos informativas, dado o número limitado de ECR completos disponíveis).

Um aspecto positivo das metanálises estudadas foi a inclusão de *preprints* (18,0%) e bases de dados nacionais (principalmente chineses; 22,5%). Estima-se que cerca de um quarto de todas as evidências relevantes sobre a COVID-19 não estejam no PubMed®, mas sim em *preprints*,(20) e a não inclusão delas representa um claro viés de publicação. O mesmo se aplica à inclusão de bases de dados nacionais, já que quantidade razoável de dados sobre epidemiologia e descrição clínica só estão disponíveis em chinês.

O número total de metanálises aumentou consideravelmente nos últimos anos,^(^^21^^)^ e essa publicação em massa tem sido fortemente criticada.(22,23) Entre os principais problemas relacionados ao aumento do número de metanálises publicadas estão esforços duplicados, resultados conflitantes, baixa qualidade e valor prático limitado.(22) Enquanto esta pesquisa se concentrou na análise da qualidade dos estudos, pode-se esperar que esses mesmos problemas também estejam presentes nas metanálises relacionadas à COVID-19. Um exemplo de resultados conflitantes em metanálises sobre a COVID-19 foi em relação ao tabagismo. Metanálise publicada em maio de 2020 concluiu que o tabagismo não representa fator de risco para casos graves de COVID-19.(24) Entretanto, duas metanálises subsequentes destacaram erros metodológicos na análise ori­ginal e chegaram à conclusão oposta.(16,25) Desde então, várias outras revisões sistemáticas foram produzidas sobre tabagismo, incluindo uma recente, que utilizou métodos robustos (uma revisão dinâmica com metanálise bayesiana, atualmente na sétima edição).^(^^26^^)^ As conclusões reforçam a complexidade dessa associação, pois constataram que fumantes atuais apresentam risco reduzido de infecção pelo coronavírus da síndrome respiratória aguda grave 2 (SARS-CoV-2), mas ex-fumantes têm maior risco de quadro grave e mortalidade por COVID-19. Apesar do número crescente de metanálises sobre inconsistências da COVID-19, a baixa qualidade e as deficiências metodológicas dessas análises podem reduzir sua capacidade de produzir resultados aplicáveis. Isso é especialmente importante para os formuladores de políticas e gestores de serviços de saúde, que podem querer usar as metanálises para orientar suas práticas e políticas.

A ideia de que as metanálises representam inequivocamente o mais alto nível de evidência médica está ultrapassada,(27) e essa afirmação certamente não pode ser aplicada a todas as metanálises. Na verdade, metanálises fracas ou imprecisas produzem menos evidências que, por exemplo, um grande estudo observacional bem conduzido ou um estudo multicêntrico controlado adequadamente. Com base nessas considerações e nos resultados dessas revisão, podem ser feitas as seguintes recomendações aos diferentes interessados sobre como interpretar as metanálises sobre COVID-19.

Para os formuladores de políticas: as metanálises são inegavelmente importantes para os formuladores de políticas, para que possam elaborar e implementar políticas usando informações confiáveis e baseadas em evidências. Porém, as metanálises atuais sobre COVID-19 têm qualidade limitada e, em alguns casos, apresentam resultados discrepantes. Portanto, em tempos de evidências incertas, a tomada de decisões não deve depender cegamente das metanálises, mas de uma análise profunda da literatura e das evidências nela contidas.

Para pesquisadores e autores de metanálises: novas metanálises sobre COVID-19 são bem-vindas, desde que forneçam novas evidências de alta qualidade. Metanálises de baixa qualidade (prematuras, redundantes, dúbias, inconclusivas ou realizadas com práticas subótimas) são contraproducentes, pois prejudicam a síntese adequada de evidências. Assim, sugere-se que futuros autores de metanálises realizem uma avaliação crítica e séria da necessidade de suas metanálises. Os fatores a seguir devem ser avaliados: se existem dados suficientes para realizar uma metanálise; se os dados disponíveis parecem confiáveis, comparáveis e livres de heterogeneidade e se já foram publicadas outras metanálises ou registrados protocolos. Os pesquisadores devem realizar uma busca por registros de protocolos de revisões sistemáticas e metanálises, para evitar esforços duplicados e redundantes. A qualidade e a robustez metodológica não devem ser negligenciadas em favor da pressa de publicar, pois isso aumenta a chance de se produzir uma revisão de baixa qualidade (conforme visto nestes resultados).

Para periódicos e editoras: as metanálises geralmente geram uma quantidade razoável de citações e boa visibilidade para os periódicos que as publicam.(28) Por isso, na situação atual, os periódicos podem se sentir tentados a publicar metanálises, ignorando métodos falhos e procedimentos de baixa qualidade. É interessante observar que, conforme o número de artigos sobre COVID-19 aumentou, cresceu também o de artigos retirados ou corrigidos.(29) Os periódicos devem cumprir seu papel social de divulgação da ciência baseada em evidências. Discussões recentes destacaram o papel dos periódicos na promoção da publicação de metanálises de alta qualidade, desencorajando, ao mesmo tempo, publicações que não sigam padrões metodológicos ideais.(30) Isso é especialmente relevante na situação atual. A revisão por pares pode ser abreviada, desde que sua qualidade e equidade não sejam prejudicadas. Sugere-se que um pesquisador com experiência em metanálises seja sempre incluído como revisor, ou que o conselho editorial inclua um especialista em estatística, a fim de garantir que metanálises com falhas ou baixa qualidade não sejam publicadas.

Alguns pontos precisam ser considerados para uma interpretação adequada deste estudo. Esta é uma revisão de escopo, e não uma revisão sistemática. Portanto, em vez de produzir nossa própria metanálise, nossa revisão buscou descrever o panorama das metanálises atuais sobre COVID-19, focando no quadro geral desta área de pesquisa, e não nos aspectos individuais de cada estudo. Decidiu-se excluir revisões sistemáticas sem metanálises. Isso porque nosso foco esteve na síntese de evidências, que são bastante limitadas em revisões que não incluem metanálises, e também, em muitos casos, o termo “revisão sistemática” é mal utilizado.(31) No entanto, uma enorme quantidade de revisões sistemáticas foi publicada, cuja qualidade já foi avaliada em um artigo anterior.(32) Esta é uma revisão com restrições de tempo. No futuro próximo, metanálises sobre a maioria dos aspectos da COVID-19 devem se tornar viáveis. Ainda assim, um estudo focado na avaliação da qualidade das metanálises publicadas até o momento sobre COVID-19 é oportuno, e este apelo pela priorização de metanálises de alta qualidade faz-se importante.

## CONCLUSÃO

As metanálises são uma ferramenta importante na medicina baseada em evidências. O número de metanálises sobre a COVID-19 está e continuará aumentando. Acredita-se que as metanálises terão um papel importante no estudo da COVID-19, definindo as características epidemiológicas da doença e os tratamentos mais adequados e apoiando a elaboração de políticas. No entanto, apesar de se concordar sobre a necessidade de metanálises sobre a COVID-19, isso não deve ser tomado como carta branca para que sejam produzidas indiscriminadamente.

Embora já tenham sido publicadas algumas metanálises bem conduzidas sobre a COVID-19, a maioria, até agora, é de má qualidade e provavelmente resultarão em mais danos do que benefícios. Poucas de todas as metanálises sobre a COVID-19 em nossa revisão poderiam ser consideradas de alta qualidade, enquanto mais da metade foi avaliada como de qualidade muito baixa. Assim, todas as partes interessadas envolvidas na pesquisa e publicação sobre a COVID-19 (incluindo formuladores de políticas, pesquisadores, editoras e pe­riódicos) devem se concentrar na pesquisa baseada em evidências, apoiando metanálises bem desenhadas, que sejam produzidas apenas quando as informações de base forem adequadas, e desencorajando as de baixa qualidade ou que utilizem métodos subótimos.
